# Does Multicomponent Physical Exercise Training Work for Dementia? Exploring the Effects on Cognition, Neuropsychiatric Symptoms, and Quality of Life

**DOI:** 10.1177/08919887221149152

**Published:** 2022-12-27

**Authors:** Flávia Borges-Machado, Laetitia Teixeira, Joana Carvalho, Oscar Ribeiro

**Affiliations:** 1 CIAFEL - Research Centre in Physical Activity, Health and Leisure, Faculty of Sports, University of Porto, Porto, Portugal; 2 Faculty of Sports, 26706 University of Porto, Porto, Portugal; 3 ITR – Laboratory for Integrative and Translational Research in Population Health, Faculty of Sports, University of Porto, Porto, Portugal; 4 ICBAS - Institute of Biomedical Sciences Abel Salazar, University of Porto, Porto, Portugal; 5 RISE – Health Research Network, ICBAS, University of Porto, Porto, Portugal; 6 CINTESIS - Center for Health Technology and Services Research, University of Aveiro and ICBAS-UP, Portugal; 7 Department of Education and Psychology, University of Aveiro, Aveiro, Portugal

**Keywords:** neurocognitive disorder, physical activity, physical exercise, cognitive function, multimodal

## Abstract

**Objective:**

To explore the effects of a multicomponent training (MT) physical exercise intervention in the cognitive function, neuropsychiatric symptoms, and quality of life of older adults with major neurocognitive disorder (NCD).

**Methods:**

Quasi-experimental controlled trial. Thirty-six individuals (25 female) were equally distributed to an exercise group (aged 74.33 ± 5.87 years) or a control group (aged 81.83 ± 6.18 years). The Alzheimer’s Disease Assessment Scale – Cognitive (ADAS-Cog), the Neuropsychiatric Inventory (NPI) and the Quality of Life – Alzheimer’s Disease (QoL-AD) tests were performed before and after the intervention.

**Results:**

There was no clear interaction effect factor of intervention on ADAS-Cog (B = 1.33, 95% CI: -2.61 – 5.28, *P* = .513), NPI (B = −8.35, 95% CI: −18.48 – 1.72, *P* = .115), and QoL-AD (B = 2.87, 95% CI: .01 – 5.73, *P* = .058).

**Conclusions:**

The 6-month MT physical exercise intervention did not present evidence of slowing down cognitive decline neither improving neuropsychiatric symptomatology, and quality of life of older adults with major NCD. Future studies with larger samples are needed to better understand the impact of physical exercise interventions using MT methodology on specific cognitive abilities, neuropsychiatric symptoms, and quality of life domains.

## Background

Along with the decline in cognitive function that characterizes a neurocognitive disorder (NCD), individuals may suffer from the progressive severity of behavioral and psychological symptoms (e.g., agitation, apathy, anxiety, hallucinations, eating disorders, nighttime behaviors) that often lead to a lower quality of life.^1^

Physical exercise, as a promising non-pharmacological therapeutical approach within dementia care^2-4^ is regularly recommended given its potential positive effects on cognitive function,^5-9^ neuropsychiatric symptomatology,^10,11^ and quality of life^12^ of individuals with major NCD.

However, according to previous systematic reviews and meta-analytic studies, it remains unclear whether physical exercise might slow down the decline of global cognition or improve specific cognitive domains (i.e., executive functions, complex attention, memory, among other abilities).^13-16^ Similarly, the role of physical exercise on alleviating challenging behaviours and mood disturbances is still limited^17-20^ particularly in what regards other symptoms rather than depression.^16,21,22^ Also, there’s still insufficient and inconsistent data on the effectiveness of well-promising physical exercise interventions on quality of life.^23-26^

Multicomponent training (MT) (i.e., a combination of balance, strength, aerobic, gait and physical function training)^27^ has been recently recommended for older adults with Alzheimer’s disease^2,28,29^ and would seem advisable to individuals with major NCD due to other etiological conditions (e.g., vascular disease). Nevertheless, despite its positive influence on functional capacity,^30,31^ few studies have focused on its effects on psychosocial outcomes. This study aims to explore the contribution of a 6-month community-based MT physical exercise intervention on the cognitive function, neuropsychiatric symptomatology, and quality of life of older adults diagnosed with major NCD.

## Methods

### Study Design and Setting

The present study takes part in the “Body & Brain” project,^32^ a two-arm quasi-experimental controlled trial with a parallel design. This project was conducted between September 2018 and May 2019 and carried out in multiple sites in the Oporto Metropolitan Area (North of Portugal).

### Participants Eligibility Criteria

Individuals aged ≥ 60 years, clinically diagnosed by a physician with major NCD or dementia (for at least 6-months) and capable of walking independently without an assistive device or human assistance were considered eligible to participate in the study. Exclusion criteria included: i) diagnosis of any condition or disorder in which exercise is contraindicated (e.g., ongoing, or unstable cardiovascular, respiratory and/or musculoskeletal condition); ii) recent hospitalization (e.g., previous month to recruitment) and/or in rehabilitation, and/or in recovery from surgery; iii) advanced stage of major NCD or dementia (e.g., ≤ 10 points on Mini-Mental State Examination [MMSE]). After agreeing to participate, eligible participants were allocated to a 6-month MT intervention – experimental group (EG), or to a social activity group – control group (CG), according to their availability.

### Interventions

EG was assigned to a 6-month group-based MT intervention (two sessions per week, 60 minutes per session) involving 5 to 8 participants. Exercise sessions were divided into 3 main parts: 10 minutes of warm-up (e.g., joint mobilization, general activation, and slow walking); 35-45 minutes of specific MT training (e.g., combining coordination and balance, lower and upper body strength, and aerobics) at light-to-moderate intensity; and five minutes to cool-down (e.g., stretching, mobility and breathing exercises). Proprioceptive and balance training included exercises with reduced sensory input, progressively difficult postures, dynamic movements, and exercises requiring stressing postural muscle groups (e.g., tandem walks, hell-to-toe stands and walks, and step over objects). Simple coordination exercises were performed (e.g., agility ladder drills). Resistance training included 4 to 6 multi-joint exercises involving major muscle groups (e.g., squats, biceps arm curl, triceps extension, chest press, seated row, standing leg curl). Aerobic endurance included low-impact exercises such as walking, dancing, and stationary marching.

Participants in the CG had a monthly social activity session (60 minutes each for 6-months, involving 5 to 12 individuals) including physical activity (e.g., breathing and relaxation activities) and the provision of information on health-related topics (e.g., falls prevention, healthy eating habits) as a complement to standard care. CG participants were contacted via phone calls to ensure retention and motivation. More information on the “Body & Brain” project study protocol can be found elsewhere.^32^

### Data Collection

Data collection occurred at baseline and after the 6-months intervention. A cutoff of 70% was considered for the adherence rate (i.e., calculated by dividing the number of attended sessions by the total number of sessions offered).

### Outcome Measures

Evaluations were conducted by specialized and well-trained researchers, who were not responsible for implementing the MT program or the social activity sessions, and were not masked to group assignment.

Participants’ sociodemographic information (age, sex, marital status, education, and living situation), and general clinical data were gathered via questionnaire.

The Alzheimer’s Disease Assessment Scale - Cognitive (ADAS-Cog)^33,34^ was used to examine individuals’ global cognitive function; it includes 11 tasks targeting memory, praxis, and language. Scores range from zero to 68, with higher scores indicating greater severity of cognitive impairment.

The Neuropsychiatric Inventory (NPI)^35,36^ was used to determine the frequency and severity of behavioural and psychological symptoms. Scores range from zero to 144 points, with higher scores suggesting worsening of neuropsychiatric symptoms.

The Quality of Life – Alzheimer’s Disease (QoL-AD),^37,38^ includes the individuals’ and caregivers’ reports on how the person with major NCD feels about 13 domains such as physical health, memory, relationship with family members, and financial situation. Scores range from 13 to 52, with higher scores indicating less impairment.

### Statistical Analyses

Data normality was verified using the Shapiro-Wilk test. Measures of central tendency and dispersion were used as appropriate to describe sample characteristics. Between groups comparisons at baseline were performed with both independent t-test and Mann-Whitney U test for continuous variables, and Pearson’s Chi-Squared test or Fisher’s Exact test for categorical variables. Longitudinal changes in the outcome measures from baseline to 6-months were analyzed using linear mixed-effects models, considering group, time, and interaction time*group as fixed effects and participant as random effect. Adjustments were performed for age and sex. The least square mean within-group difference was estimated from these models. A significance level of *α =* .*05* was established. All statistical procedures were carried out with SPSS IBM Statistical version 27.0 and with R software version 4.0.4.

## Results

### Participants

**Figure 1.**
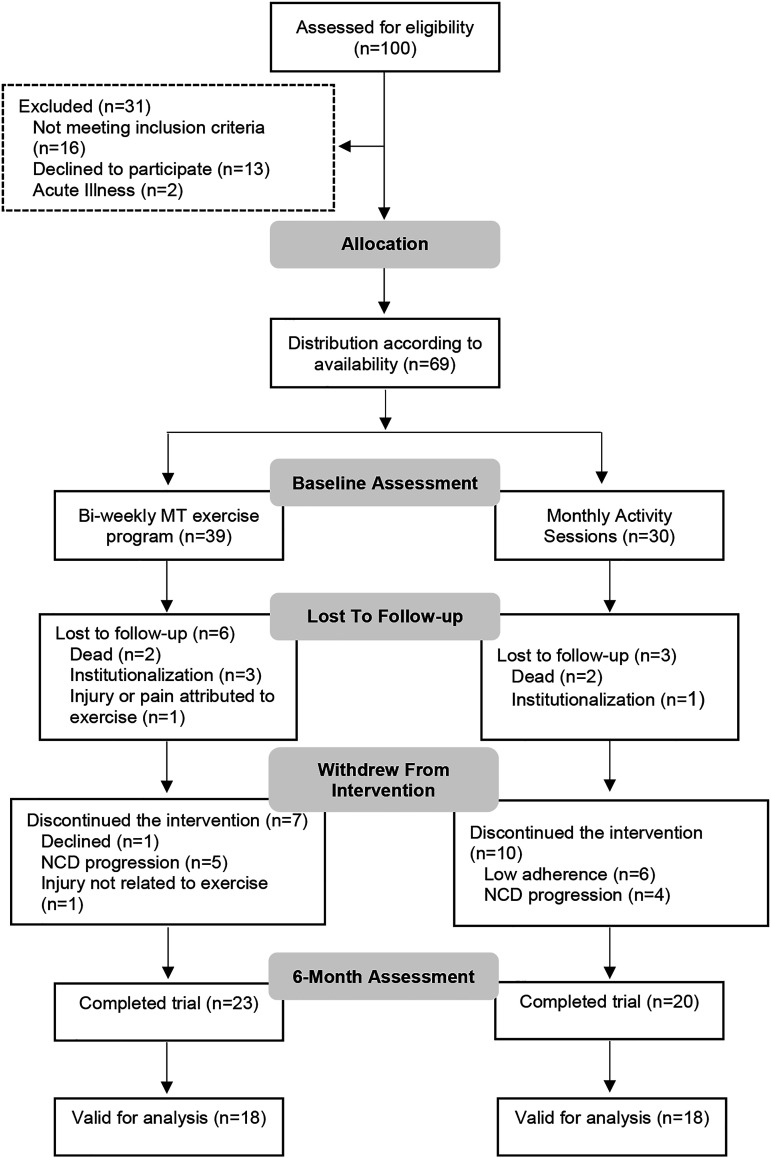
Study Flow Diagram.

From the 69 individuals diagnosed with NCD (mean 79.50 years old (SD = 6.73), 72.5% female) included in the “Body & Brain” project, 26 were lost to follow-up or discontinued the intervention (dropout rate of 38%). Forty-three participants (mean 78.26 years old (SD = 6.68), 69.8% female) completed the intervention: 23 in the EG and 20 in the CG. Seven participants (5 from the EG and 2 from the EG) presented minor NCD and, for this reason were not considered valid for this study analysis. The flow-chart of participants during the trial is shown in Figure 1.

Thirty-six older adults (mean 78.08 years old, SD = 7.05) were included in the present study, distributed in equal number between both groups. Twenty individuals (55.6 %) had an unspecified diagnosis of major NCD or dementia, 8 (22.2 %) due to Alzheimer’s disease, 3 (8.3 %) due to multiple etiologies, 3 (8.3 %) due to Parkinson’s disease, 1 (2.8 %) due to Lewy bodies disease and 1 (2.8 %) due to Korsakoff syndrome. Within both groups, major NCD or dementia diagnosis were established for at least 6 months and a maximum of 12 years. Globally, individuals presented a baseline mean score on MMSE of 20.47 (SD = 4.91) points. More than 80% of the individuals had a prescription at the beginning of the intervention of, at least, one psychopharmacological drug. Specifically, 29 (80.6 %) participants were taking antidepressants, 12 (33.3 %) were taking hypnotics, sedatives, or anxiolytics and 7 (19.4 %) were taking antipsychotics. Twenty-five participants (69.4 %) were female, half were widowed (50.0 %), and almost half had at least 4 years of formal education (45.7 %). Most individuals (72.2 %) lived in the domiciliary context with their spouses or other family members (i.e., children, nephew). As presented in Table 1, participants’ baseline characteristics did not significantly differ between the groups, except for age and number of diagnosed chronic comorbidities.

**Table 1.** Baseline Sample Characteristics.

Characteristics	Total (*n* = 36)	EG (*n* = 18)	CG (*n* = 18)	Statistical Inference
Age (years), mean (SD)	78.08 (7.05)	74.33 (5.87)	81.83 (6.18)	*P* < .001^a^
Age, range	61-89	61-83	70-89	
Sex (female), n (%)	25 (69.4)	13 (72.2)	12 (66.7)	*P* = .727^b^
Civil Status, n (%)				
Widow	18 (50.0)	7 (38.9)	11 (61.1)	*P* = .357^c^
Married or Civil Union	15 (41.7)	10 (55.6)	5 (27.8)	
Divorced or Separated	3 (8.3)	1 (5.6)	2 (11.1)	
Years of Formal Education, mean (SD)	4.00 [4.47-6.44]	4.00 [4.32-7.46]	4.00 [3.69-6.31]	*P* = .463^d^
Education Levels, n (%)				
Low (1-3 years)	9 (25.7)	4 (22.2)	5 (29.4)	*P* = 0.830^c^
Medium (4-6 years)	16 (45.7)	8 (44.4)	8 (47.1)	
High (7-12 years)	10 (28.6)	6 (33.3)	4 (23.5)	
Living Situation, n (%)				
With Spouse or Other Family Member	26 (72.2)	13 (72.2)	13 (72.2)	*P* = 1.000^c^
Nursing Home	5 (13.9)	2 (11.1)	3 (16.7)	
Alone	5 (13.9)	3 (16.7)	2 (11.1)	
Number of Medications and Supplements, mean (SD)	7.00 [6.46-8.71]	8.00 [6.60-10.40]	6.50 [5.41-7.92]	*P* = .226^d^
Number of Comorbidities, mean (SD)	3.81 (2.04)	4.50 (2.07)	3.11 (1.81)	*P* = .039^a^
Disease Duration (years), mean (SD)	3.00 [2.21-3.89]	3.00 [2.16-4.67]	2.00 [1.43-3.57]	*P* = .285^d^
Dementia or Major NCD Diagnosis, n (%)				
Unspecified	20 (55.6)	9 (50.0)	11 (61.1)	*P* = .748^c^
Alzheimer’s Disease	8 (22.2)	5 (27.8)	3 (16.7)	
Multiple Etiologies	3 (8.3)	1 (5.6)	2 (11.1)	
Parkinson’s Disease	3 (8.3)	1 (5.6)	2 (11.1)	
Lewy Bodies Disease	1 (2.8)	1 (5.6)	0	
Korsakoff Syndrome	1 (2.8)	1 (5.6)	0	

*Notes*: Values are presented as mean (SD) (continuous variables), median [interquartile range] or percentage (categorical variables)

*MMSE* Mini Mental State Examination, *NCD* Neurocognitive Disorder

Missing: 1 – Education Levels, Years of Formal Education; 6 – Disease Duration

^a^t – Independent sample T-test

^b^χ2 – Pearson’s Chi-Square test

^c^Fisher’s Exact test

^d^U – Mann-Whitney U test.

No serious adverse events occurred during the intervention. Considering the total number of sessions within the 6-months, adherence to the bi-weekly exercise sessions was higher than 75% (mean 87%).

### Outcomes

As presented in Table 2, a significant interaction (time X group) effect factor was not found for none of the variables. But age was a significant effect factor on NPI test (B = −.64, 95% CI: −1.17-−.11, *P* = .029).

**Table 2.** Linear Mixed Models for Cognitive Function, Neuropsychiatric Symptoms, and Quality of Life.

	Linear Mixed Effects Adjusted for Age and Sex												
	Fixed Effects									Random Effects			
	Time			Group			Time X Group			ID		Residual	
	**B**	**95% CI**	***P value***	**B**	**95% CI**	***P value***	**B**	**95% CI**	***P value***	**SD**	**95% CI**	**SD**	**95% CI**
*ADAS-Cog*	0.28	−2.51-3.07	0.847	−1.38	−8.97-7.64	0.795	1.33	−2.61-5.28	0.513	12.22	9.10-14.96	4.28	3.35-5.34
*NPI* ^a^	9.07	1.90-16.30	0.019	−1.00	−9.73 – 7.74	0.829	−8.35	−18.48 – 1.72	0.115	4.69	0.00-8.21	10.87	8.50-13.25
*QoL-AD*	−2.57	−4.60-−0.55	0.018	−3.17	−6.15-−0.19	0.049	2.87	0.01-5.73	0.058	2.59	1.05-3.55	3.10	2.43-3.87

^a^Age was a significant factor

Group: control group is reference. Time: baseline is reference

*ADAS*-Cog Alzheimer’s Disease Assessment Scale – Cognitive, *NPI* Neuropsychiatric Inventory, *QoL-AD* Quality of Life – Alzheimer’s Disease

After a 6-month MT physical exercise intervention, the EG slightly improved the least square mean scores on QoL-AD test (Table 3), which is indicative of increased quality of life. As opposite, individuals in the CG decreased the scores in this variable. In parallel, participants in the EG revealed a slight increase in the ADAS-Cog score, indicative of greater severity of cognitive impairment, whereas the CG maintained the scores. Both groups experienced an increase in the NPI, which was more significative on CG individuals, suggesting worsening of behavioral and psychological symptoms.

**Table 3.** Within-Group Differences From Baseline in the Cognitive Function, Neuropsychiatric Symptoms, and Quality of Life.

		Experimental		Control	
		*n*	Mean (SE)^a^	*n*	Mean (SE)^a^
*ADAS-Cog*	M1	18	29.9 (3.71)	18	31.2 (3.35)
	M2	18	31.5 (3.71)	18	31.5 (3.35)
NPI*	M1	18	11.4 (3.19)	17	12.4 (3.09)
	M2	18	12.1 (3.19)	18	21.5 (2.96)
*QoL-AD*	M1	18	29.8 (1.11)	18	32.9 (1.02)
	M2	18	30.1 (1.11)	18	30.4 (1.02)

^a^Least square mean calculated from linear-effects mixed models

*ADAS*-Cog Alzheimer’s Disease Assessment Scale – Cognitive, M1 Baseline, M2 After 6-month Intervention, *NPI* Neuropsychiatric Inventory, *QoL-AD* Quality of Life – Alzheimer’s Disease, SE standard error

*Significant difference within-group from baseline (*P* < .05)

## Discussion

The results from this study suggest that a 6-month MT physical exercise intervention was not effective in improving cognitive function, neuropsychiatric symptomatology, and quality of life of older adults diagnosed with major NCD. Previous studies attested MT effectiveness in improving individuals with NCD lower body function,^30,31^ gait speed and mobility.^30,39^ However, less evidence has been shown on the effects of MT physical exercise interventions on psychosocial outcomes.^40-42^

Previous meta-analytic and systematic review studies indicated that physical exercise interventions might improve cognitive function, or slow down the decline in individuals with major NCD; still, findings have been inconsistent across studies.^13-16,43^ The questionable benefit of physical exercise on cognition might be explained by the methodological differences across studies (e.g., eligibility criteria, diagnostic criteria or referral protocol, design and settings), the heterogeneity of training programs in terms of frequency, type, intensity, and duration; the significant variability in cognitive assessment tools and instruments; and by the range of subtypes/stages of major NCD.^5,6,13,16,44^

Knowing if physical exercise improves or not global cognition is important but insufficient to healthcare providers in advising this type of intervention.^10^ Recent findings showed the effects of physical exercise in specific cognitive domains, such as working memory, cognitive flexibility, attention, and executive functions^7,8,10^; but this relationship is not always true across studies.^13^ To some extent, the questionable benefit of physical exercise on cognition is attributed to the selection of evaluation measurements.^13,14,43^ The ADAS-Cog instrument, which is subdivided in 11 tasks targeting 3 cognitive functions (memory, language, and praxis), is a widely used measure and sensitive to change,^45,46^ but it does not allow to examine the effects of physical exercise on planning, decision-making, working memory, responding to feedback, inhibition and mental flexibility. The slight increment in ADAS-Cog score verified in our study in the EG after the 6-months intervention may be explained, at least in part, by the pervasiveness of unspecified subtypes and/or missing data on disease stage, and by the limitations of the assessment tool (e.g., assess of global cognition), as expressed in the existing literature.^19^ The development of a Core Outcome Set may be an effective solution to standardize the evaluation of physical exercise effects on each cognitive ability^44,47,48^ and, thereby, help the development of clear guidelines on physical exercise prescription for people with any type of major NCD.

As for the non-observed effects on neuropsychiatric symptoms, worthwhile referring is the instrument used for data collection. The NPI test is the most widely used instrument in nonpharmacologic interventions to determine its benefit on neuropsychiatric syndromes that occur in Alzheimer’s disease and other neurodegenerative disorders^11,49^; but, as underscored by Ferreira and colleagues,^50^ the caregivers’ subjective appraisal on symptoms severity may induce variability on its evaluation. For this reason, the usage of shorter frequency ratings like the Abe’s BPSD Score^51^ has been described as more prone to a direct and objective assessment by caregivers.^50^ Using it in research on MT interventions might be a possibility in further studies, following recent efforts to make an European Portuguese version of the instrument available for research.^50^ Also, controversial findings have been reported on the influence of physical exercise as a non-pharmacological first-line treatment for the management of neuropsychiatric symptoms.^17,19,52^ Kouloutbani and collaborators^11^ for instance, showed the benefits of aerobic training interventions on alleviating depressive symptomatology, but the high variability on program characteristics and inclusion of different stages of dementia did not allow these authors to draw on firm conclusions. So far, most studies have been focusing exclusively on depression^16,21,22^ and, therefore, data is sparse on other behavioural signs (e.g., aberrant motor behaviour) and psychological symptoms (e.g., apathy, anxiety, delusions, hallucinations).^17^ However, beyond changes in the NPI total score, the authors could not conduct further sub-analyses due to sample size restrictions. It is necessary, for these reasons, to further investigate the potential effects of MT interventions on alleviating specific neuropsychiatric symptoms aside from depression in individuals with major NCD from mild to severe stages through culturally validated frequency scales.

In terms of the effects of the MT physical exercise on the quality of life, our study findings showed promising results for the QoL-AD total scores, which suggests a potential positive effect. A larger and more homogenous sample (i.e., in terms of stage and subtype of dementia) could have resulted in a statistically significant interaction factor between group and time. According to Ojagbemi and Akin-Ojagbemi^24^ meta-analysis study, aerobic physical exercise seems to potentially produce a larger effect (though still not statistically significant) on quality of life when compared with non-aerobic or combined methodologies, but a number of methodological issues related to training specifications (i.e., frequency, intensity, type, and time) and dementia stage/subtype affect results interpretation. In fact, there is a lack of evidence supporting physical exercise interventions (of any training modality) for improving the quality of life and well-being in individuals with major NCD,^23^ possibly due to the limitations underlying the conceptualization and assessment of quality of life in this population.^53-55^ As a result, these empirical and theoretical challenges do not allow the transferability of the assumption that physical exercise may also improve the quality of life and well-being of individuals with major NCD just like it improves in the general older population.^23,24^ QoL-AD is a frequently used measure, particularly in studies considering both self-rated and caregiver-rated quality of life,^24,54^ but future research is still needed to understand if it is the most appropriate instrument to determine the effectiveness of physical exercise interventions. As suggested by Ojagbemi and Akin-Ojagbemi,^24^ a combination of proxy and self-reports, as well as the examination of physical functioning, may provide a more comprehensive information about the effects of physical exercise in the quality of life of people with major NCD. Furthermore, evidence is also required to attest if MT may be an effective methodology to improve quality of life and allow clear recommendations.

The quasi-experimental design and the small sample size need to be acknowledged as major limitations of our study; therefore, results should be interpreted with caution and not generalized. Due to difficulties during the recruitment process, some participants were tentatively included in the “Body & Brain” project even if their diagnosis of major NCD was still under study at baseline evaluations; for this reason, some participants who have completed the intervention were excluded due to the later confirmation of a minor NCD diagnose, increasing the already high dropout rate. Moreover, possibly as a consequence of the delayed implementation of the national strategy for dementia,^56^ also clinical data on dementia subtype or stage was scarce in the patients who were considered eligible to participate in the study, constraining the possibility of more detailed analysis. Such a lack of information has been reported in several national studies aiming to estimate dementia prevalence rates.^57,58^ Lastly, the authors want to point out that evaluators were not blind to group assignment, which can be partially justified by the nature of this intervention and sample specifications.

## Future Research Implications

Future trials should continue exploring the effects of MT training methodologies in cognition, neuropsychiatric symptoms, and quality of life, and intent to provide more detailed information on the impact MT has on specific cognitive abilities, such as executive functions, and on distinct behavioral and psychological symptoms. For such an aim, reliable measures should be considered, as the gold standard methods may not always be the best solution. MT physical exercise has been increasingly recommended for older adults with and without functional limitations,^28^ and even for those already diagnosed with Alzheimer’s disease,^2^ which justifies the need to explore the potential effect of this training methodology on different stages/subtypes of major NCD.

## Conclusion

The 6-month MT physical exercise intervention did not present evidence of slowing down cognitive decline neither improving neuropsychiatric symptomatology, and quality of life of older adults with major NCD. A MT intervention can be an effective way to slow down cognitive decline, manage challenging behaviors and improve the quality of life of individuals with NCD, but further research is necessary to attest the influence of this training methodology. There is still a long way to go in what concerns physical exercise recommendations for people with major NCD, but, in the absence of a cure or disease-modifying therapy, it is of utmost importance to explore the potential effects of validated methodologies that might slow down the disease progression or alter its course, delaying or avoiding nursing-home placement.
